# A Novel Class of Cost Effective and High Performance Composites Based on Terephthalate Salts Reinforced Polyether Ether Ketone

**DOI:** 10.3390/polym11122097

**Published:** 2019-12-14

**Authors:** Franco Dominici, Debora Puglia, Francesca Luzi, Fabrizio Sarasini, Marco Rallini, Luigi Torre

**Affiliations:** 1 Civil and Environmental Engineering Department, University of Perugia, Strada di Pentima 4, 05100 Terni, Italy; debora.puglia@unipg.it (D.P.); francesca.luzi@unipg.it (F.L.); marco.rallini@unipg.it (M.R.); luigi.torre@unipg.it (L.T.); 2 Department of Chemical Engineering Materials Environment, Sapienza-Università di Roma and UdR INSTM, Via Eudossiana 18, 00184 Roma, Italy; fabrizio.sarasini@uniroma1.it

**Keywords:** poly(ether ether ketone), PEEK, calcium terephthalate salts, high performance nanocomposites

## Abstract

Poly(ether ether ketone) (PEEK)-based nanocomposites have been realized with incorporation (0–30 wt %) of anhydrous calcium terephthalate salts (CATAS), synthetized by reaction of terephtalic acid with the metal (Ca) oxide, by means of a melt processing. Their structure, morphology, thermal, and mechanical properties have been investigated. Scanning electron microscopy observations confirmed homogeneous dispersion of nanometer-sized fillers and a toughened fracture morphology even at the higher content, while thermal characterization confirmed an unvaried thermal stability and unmodified crystalline structure of the reference PEEK matrix. A negligible nucleating effect was evidenced, while a blocking effect of the amorphous phase fraction provide composites with increased stiffness, confirmed by enhanced values of G’ and shifts of glass transition peak to higher temperatures, for restriction in chain mobility imposed by CATAS. The proposed solutions aimed to enlarge the application range of high performance costly PEEK-based composites, by using thermally stable nanofillers with limited costs and easily controllable synthesis phase.

## 1. Introduction

Nowadays, the use of composite materials based on polymeric matrices constitutes an irreplaceable reality in the field of innovative materials. The development of polymers with exceptional properties to be used in ever-wider sectors has been accompanied by the evolution of filling/reinforcement materials suitable for making composites with improved characteristics and reduced costs [1]. Thanks to their exceptional characteristics, high performance polymers have progressively conquered application roles that previously were the exclusive prerogative of traditional materials; even better, composites based on these polymeric matrices attempt to increase performance, possibly even reducing costs, to expand the range of high-performance and cost-effective applications.

The usefulness of nanoreinforcements has a double advantage: both for the performance aspect and from an economic point of view, contributing to lower costs and expanding the market for this class of materials [2]. Polyether ether ketone (PEEK) is a high performance semi-crystalline thermoplastic polymer that offers a unique combination of mechanical properties, chemical resistance, wear, fatigue and creep, as well as exceptionally high temperature resistance in its composites up to 260 °C. The polymer belongs to the family of polyarylether ketone polymers (PAEKs), characterized by phenylene rings that are linked via oxygen bridges giving heat and mechanical resistance; among these, PEEK is the most widely used and produced on a large scale, but its main restraining factor is the cost, which is currently around 50/70 $/kg. However, the identification of reinforcements suitable for the production of thermoplastic composites with high temperature matrices as PEEK is rather complex [3]. There are many problems that must be faced to satisfy the prerogatives of using fillers. The main characteristics to be found can be summarized as thermal stability, physical-chemical compatibility with matrices, nanometric size, and low cost. In order to have a good productivity, continuous processes must be used, such as extrusion, and the method used to produce the composites turns out to be melt blending; for this reason, the reinforcements must possess thermal stability up to the characteristic process temperatures of each matrix for the time necessary to the dispersion in the melt, usually of 370–400 °C for 2–3 min.

However, traditional compatibilization treatments used for reinforcements in thermoplastic matrix composites (e.g., silanization or maleic grafting) cannot be applied due to thermal degradation. According to this, research must therefore be directed toward nanoreinforcements that can be mixed with matrices at high temperatures, capable of forming good quality bonds without losing effectiveness and having a competitive cost, that cannot actually be obtained by using ceramics, such as Si_3_N_4_ [4] or boron nitride [5], carbon nanotubes [2], zinc, or titanium oxides [6,7] for use under extreme mechanical and tribological loads.

Terephthalate salts seem to satisfy all the required criteria. Moreover, there are no examples in the scientific literature of these salts used for the production of thermoplastic composites. The terephthalate salts can be obtained by the reaction of terephthalic acid with different metal oxides to obtain the terephthalate salts of the metal used in the reaction. In applications that use terephthalate salts for making batteries, it has been shown that nanometer crystals are used [8]. The production of salts with nano-sized lamellar structure can be obtained with appropriate controls of the reaction parameters [9]. Many different terephthalate salts, sodium, potassium, aluminum, magnesium, calcium, and other metal salts may be obtained [10,11]. Many of these salts show good thermal stability in the range of temperatures characteristic for the extrusion process of the matrices under study. Panasyuk et al. demonstrated, with thermogravimetric analysis performed on some terephthalate salts, good thermal stability below 400 °C [12]. The characteristic benzene ring suggests that there may be good compatibility between the terephthalate salts and the polymer molecules for high temperature applications, which are also rich in stable aromatic rings. Furthermore, the bound water molecules of some salts, such as magnesium, calcium, and aluminum salts, can probably offer further bonds if properly removed [13].

In the present work, we intend to investigate the possibility of using calcium terephthalates to make composites based on polyether ether ketone (PEEK) with improved characteristics. Main aims of the study are improvements in thermomechanical properties and cost reduction. The pursued improvements consist in enhancing the thermo-mechanical properties that could widen the range of applications of high performance composites (HPC); likewise, it is also important to reduce the cost through the addition of cheap fillers that would make the market for HPC, such as these PEEK-based materials, affordable for a wider range of industrial sectors.

## 2. Materials and Methods

A commercial grade of polyether ether ketone (trade name, Vestakeep 2000P, Evonik Degussa GmbH, Essen, Germany) was supplied by Evonik. This PEEK is characterized by a density of 1.30 g/cm^3^ at 23 °C (ISO 1183) and a melt volume-flow rate (MVR) of 70 cm³/10 min at 380 °C/5 kg (ISO 1133). A nanometric metal-organic framework consisting of calcium ions as metal clusters coordinated to terephthalic acid as organic ligand was synthesized in our laboratories to be used as filler in the composites. Chemical reagents calcium oxide, terephthalic acid, ammonia and water, were supplied by Sigma Aldrich (Milan, Italy). The synthesis method is briefly described in the next section.

### 2.1. Preparation of Terephthalate Salts

Powdered terephthalic acid (TPA) was solubilized in an alkaline solution of water and ammonia with stirring at 80 °C for 1 h. Then the calcium oxide was added in a stoichiometric proportion to the acid, according to the reaction (1):

C_8_H_6_O_4_ + CaO + 2 H_2_O → C_8_H_4_O_4_Ca • 3 H_2_O (1)

The solution was kept under stirring at 80 °C for 30 min, where the insoluble calcium terephthalate precipitated forming a whitish deposit on the bottom. The solution was filtered, and the solid residue subjected to appropriate washings to eliminate unwanted reaction residues. At the end of the synthesis, calcium terephthalate trihydrate is obtained. In order to eliminate both moisture and water-bound molecules, the product was kept in a vacuum oven for 2 h at 190 °C. Finally, the anhydrous calcium terephthalate (CATAS) was pulverized and sieved to eliminate any conglomerates of salts.

### 2.2. Production of Composite Materials

Composite materials were manufactured using the melt mixing method by blending the PEEK polymer matrix with the CATAS organic metal fillers as shown in Table 1. A co-rotating twin-screw extruder, Microcompounder (DSM explorer 5&15 CC MicroCompounder, Xplore Instruments BV, Sittard, The Netherlands) 5 and 15cc by DSM, coupled to a press, Micro Injection Molding Machine 10cc (DSM explorer 5&15 CC MicroCompounder, Xplore Instruments BV, Sittard, The Netherlands), were used for the production of the samples. The good thermal stability of the materials allowed to mix for 5 min at 100 rpm by setting a temperature profile of 355–375–390 °C in the three heating zones from feeding to die; an adequate pressure profile was used for the injection while barrel and mold temperatures were set at 395 and 190 °C, respectively. The samples produced were subjected to subsequent characterizations.

### 2.3. Characterization of Terephthalate Salts and PEEK/CATAS Composites

Morphological characterization of salts and PEEK_CATAS composites was carried out using a field emission scanning electron microscope (FESEM) Supra 25 by Zeiss (Oberkochen, Germany) taking micrographs with an accelerating voltage of 5 kV at different magnifications. Previously, the samples were gold sputtered to provide electric conductivity.

X-ray diffraction (XRD) analysis was performed with a diffractometer X’Pert PRO by Philips (Malvern Panalytical Ltd, Malvern, UK)(CuKα radiation = 1.54060 Å) at room temperature. XRD patterns were collected in the range of 2θ = 10°–80° with a step size of 0.02° scan and a time per step of 34 s.

Fourier transform infrared (FT-IR) spectra were recorded using a Jasco FT-IR 615 spectrometer (JASCO, Easton, MD, USA) in the 400–4.000 cm^−1^ range, in transmission mode. The CATAS materials were analyzed using KBr discs made by mixing terephthalate salts and KBr powder.

Thermogravimetric analysis (TGA) was performed for salts and PEEK_CATAS composites with a thermobalance Exstar 6300 (Seiko, Tokyo, Japan) setting a dynamic scan with temperature ramp at a speed of 10 °C min^−1^ in the range 30–800 °C under a nitrogen atmosphere (200 mL min^−1^). Derivative of mass loss (DTG) was calculated to measure the rate of the thermodegradative phenomena.

Thermal characteristics of PEEK composites were investigated with a temperature-modulated differential scanning calorimeter (MDSC) Q200 by TA Instruments (DSC, TA Instrument, Q200, New Castle, DE, USA). A heating, cooling, and heating cycle between 25 and 400 °C was performed at a rate of 10 °C min^−1^ in nitrogen flow at 60 mL min^−1^.

Dynamic mechanical behavior of the produced materials was studied using an Ares N2 rheometer (Rheometric Scientific, Epsom, Surrey, UK), samples of about 4mm × 10mm × 40 mm, gripped with a gap of about 20 mm were tested in rectangular torsion at a frequency of 2π rad/s with a strain of 0.05%, and a temperature ramp of 3 °C/min applied in the range from 30 to 250 °C.

Rheological characterization of polymer composite melts was performed by using parallel plates geometry of Ares N2 rheometer (Rheometric Scientific), with dynamic strain frequency sweep tests isothermally performed at 400 °C with frequencies ranging from 0.1 to 100 rad/s at 10 points/decade and with a strain of 0.3%.

## 3. Results and Discussion

### 3.1. Characterization of Calcium Terephthalate Salts

**Figure 1:**
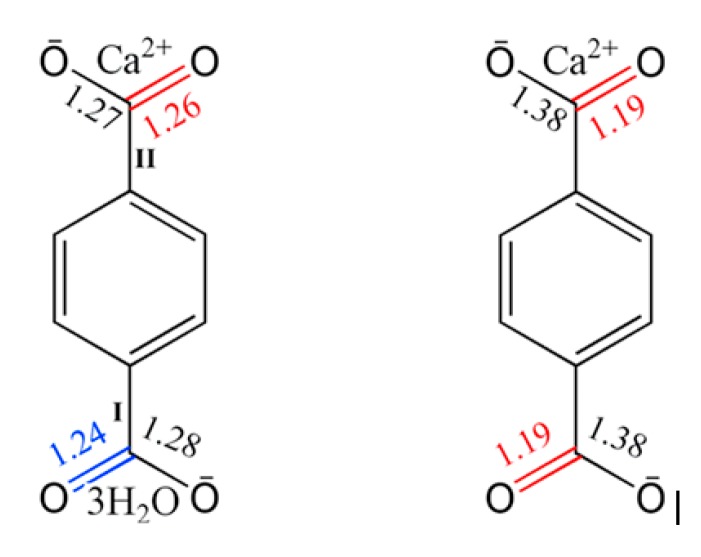
Chemical structure of calcium terephthalate salts before (**left**) and after (**right**) thermal treatment (evidence of structural rearrangement with indication of bond length) reprinted from [16].

Because of their good thermal stability above 400 °C, calcium terephthalate salts have been selected to be used as fillers in high melting polymers, such as PEEK. The possibility of removing the bound water molecules to form bonds with the polymeric molecules and the particular structure formed by the terephthalic acid ions with the calcium ions suggests the possibility of a good compatibility, with formation of strong bonds between fillers and polymers. The structural characteristics of terephthalate calcium salts are shown in Figure 1, where the chemical bonds between the terephthalate ion, the calcium ion, and the three water molecules are highlighted [14,15].

**Figure 2:**
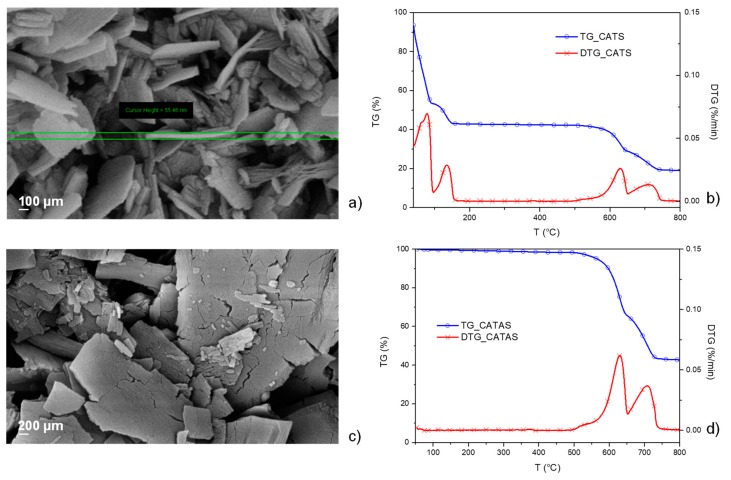
Field emission scanning electron microscope (FESEM) micrograph of calcium terephthalate trihydrate salts (CATS) (**a**) and thermogravimetric/derivative of mass loss (TG/DTG) curves for CATS (**b**); FESEM micrograph of CATAS (calcium terephthalate anhydrous salts)(**c**) and TG/DTG curves for CATAS (**d**).

The reaction between terephthalic acid and calcium oxide in water was carried out to produce trihydrate calcium salts terephthalate [17]. The production process required an appropriate monitoring of reaction conditions, by acting on the kinetics and using appropriate additives to obtain the nanometric structures. Some attempts have been made to optimize the characteristics of the fillers. Lastly, insoluble calcium terephthalate trihydrate salts (CATS) were obtained by their precipitation in water. A SEM micrograph of the salts obtained with the optimized reaction parameters is shown in Figure 2a. Morphologies of CATS is characterized by a plate shape with smooth surface and a nanometric size of the lamella thickness of about 50 nm.

In order to evaluate the thermal stability and compare the results with the values found in the literature, a thermo-gravimetric analysis was performed on the obtained salts. The salt sample was tested with dynamic TGA and the results of mass loss (TG) and derivative weight loss (DTG) are shown in Figure 2b. The four mass loss zones shown in the TG curve correspond to the four peaks highlighted in the DTG curve. The first peak, at a temperature lower than 100 °C, refers to the loss of hygroscopic water and any residual volatile additives present during the reaction. The second peak, at a temperature between 100 °C and 170 °C, refers to the loss of molecular water since a weight loss of 22% (moisture free weight) corresponds to three water molecules per formula unit. The decomposition of the acid salts involved the breaking of the carboxyl groups, the formation of carbonates, and the release of gaseous products. The subsequent mass losses are due to thermo-oxidative destruction of organic ligands and subsequent oxidation of the residue over 500 °C [18]. Since the TG analysis showed that a heat treatment above 170 °C transforms the trihydrate salts into the corresponding anhydrous calcium terephthalate (CATAS) salts, the hydrated salts were heated at 200 °C for 1 h in a vacuum oven. The presence of bound water could be harmful for the melt blending process of the composites, giving rise to hydrolytic phenomena and formation of cavities within the samples. Figure 2c shows that, after the dehydration (*T* = 200 °C), the anhydrous salts have cracks with rough surface, revealing that volume contraction happens in the dehydration process [16].

In Figure 2d, thermogravimetric analysis of the CATAS shows the absence of relative humidity and water molecular peaks below 170 °C, because of an effective dehydration treatment. The thermal stability at the process temperatures of the composites (355–395 °C) is confirmed by the substantial absence of peaks in the DTG. Degradation phenomena are noted above 500 °C as for hydrated salts.

**Figure 3:**
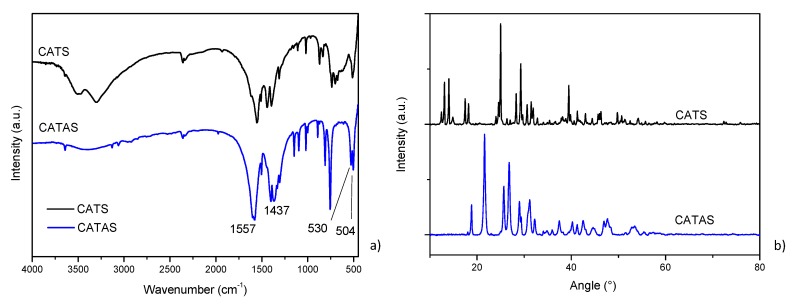
Fourier transform infrared (FT-IR) (**a**) and X-ray diffraction (XRD) (**b**) spectra of CATS and CATAS.

FTIR measurements (Figure 3a) before and after dehydration confirmed the results already reported in the literature: absence of characteristic absorption peaks of terephthalic acid (e.g., 1671 and 1420 cm^−1^) demonstrates a complete reaction in our synthesis procedure. Strong bands at 1557 and 1437 cm^−1^ are assigned to asymmetric and symmetric stretching vibration of carbonyl groups, respectively. Bending vibration of (Ca–O) presents a band at 515 cm^−1^. The bands of (Ca–O) in CATS 3H_2_O at 515 cm^−1^ splits into double peaks (530 and 504 cm^−1^) in CATAS, presumed that the coordination environment for Ca^2+^ is changed [8,16,19].

The X-ray diffraction pattern of CATAS shows the reflections corresponding to CATS, which are shifted toward higher angles (Figure 3b). Mazaj proposes a reversible hydration/dehydration mechanism that through the breaking and reconstitution of Ca–O bonds deforms the crystalline structure generating the displacement of the reflection angles. In particular, the process involves the rotation of the terephthalate ligand, disconnection and protonation of the carboxylate group on one side and the partial disconnection and reformation of the Ca–O bond on the other side of the ligand [19]. The TPA ligand that is connected to four CaO_6_ chains in a monodentate way of the CATAS structure connects to a CaO_8_ chain in a bidentate manner, with the carboxylate that becomes protonated and COO– group turns into ionized. XRD pattern of CATAS shows that significant diffraction peaks appear at 18.8°, 21.6°, 25.7°, 26.9°, and 31.2°. According to the report by Mou et al. this structure can be indexed with a space group of C 2/c (n. 15) as monoclinic crystal system [8].

### 3.2. Characteristics of Composites PEEK/Calcium Terephthalate Salts

**Figure 4:**
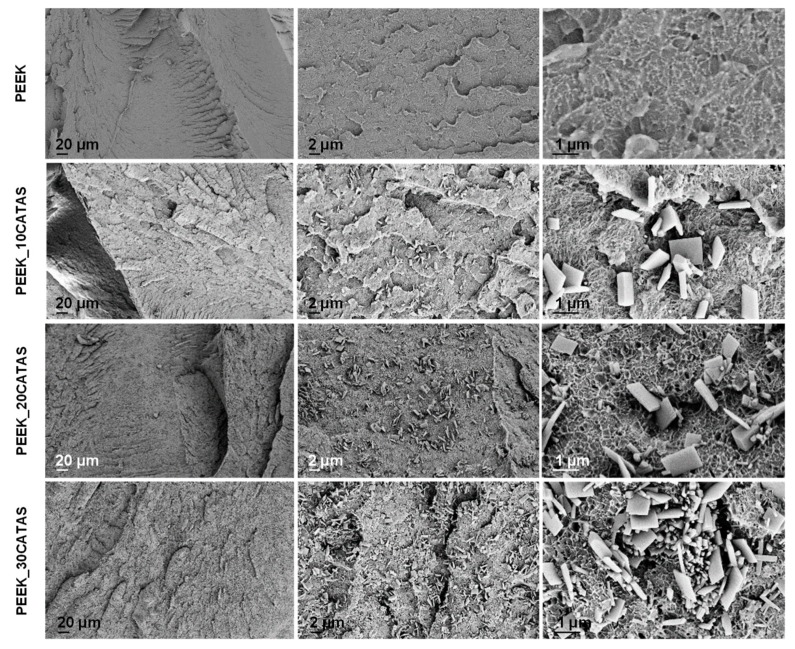
FESEM micrographs of neat PEEK and PEEK nanocomposites with 10, 20, and 30 wt % of CATAS at different magnification.

Supposing good compatibility between matrix and filler, four materials were produced via melt blending by adding 0%, 10%, 20%, and 30% by weight of CATAS to PEEK matrix (Table 1). The salts were added to the mixture, taking care to avoid rehydration. Samples of 4mm × 10mm × 80 mm were injection molded, sized as necessary, and used for characterization. FESEM morphology of the fractured surfaces in liquid nitrogen for the composite materials was observed. Figure 4 shows micrographs at three magnifications for each material.

Micrographs show uniform fracture surfaces with homogeneous dispersion of nanometer-sized fillers. Observing the micrographs, we noted that all the materials show a toughened fracture morphology. This aspect is also noted at higher magnification, where the typical flake conformations with plasticized peduncles confirm a tough behavior of the materials [20]. A wide heterogeneity of salts is observed, probably because of a milling effect during compounding; however most of the fillers still have a lamellar appearance. Despite the progressive increase in the quantity of nanofillers in the three composites, reaching a good degree of filling with 30 wt % of CATAS, the toughened fracture morphology suggests that even the high filled composite has not reached full saturation.

**Figure 5:**
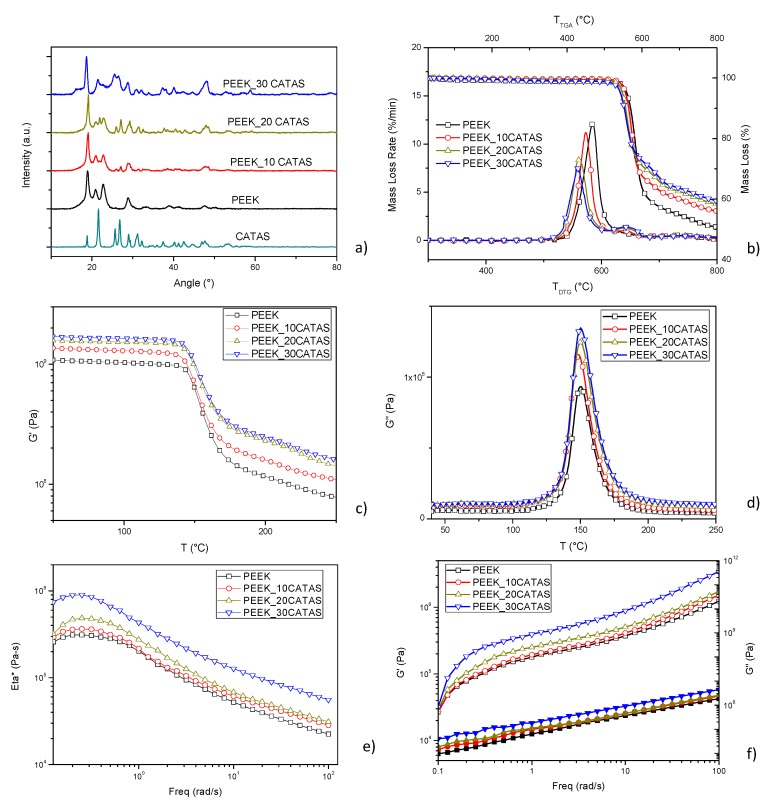
(**a**) XRD patterns; (**b**) TG/DTG curves; (**c**) G’ and (**d**) G” curves; (**e**) complex viscosity; and (**f**) storage (open symbol) and loss moduli (closed symbol) at 400 °C for PEEK and PEEK_CATAS nanocomposites at different CATAS content.

X-ray diffraction analysis performed on composites (Figure 5a) shows that the reflection peaks of the composites correspond to the normalized overlay of the PEEK and CATAS reflection patterns. The XRD pattern of PEEK matrix revealed three representative crystalline peaks indexed as (110), (200), and (020), and resolved at 18.9°, 22.8°, and 28.9°. No shifts in the main peaks are noted which could indicate changes in the crystalline structure of the composites. It should be noted that, as the filler content increases, the CATAS reflection peaks are further noticeable in the XRD pattern of the composites. It can be deduced that the CATAS do not undergo substantial changes in their crystalline structure and do not even cause changes to the crystalline phase of the polymer matrix [21,22].

The influence of the CATAS nanofillers on the thermal stability of the nanocomposites has been investigated by thermogravimetric experiments, carried out under inert conditions, from 30 to 800 °C. The TG/DTG curves of the different PEEK nanocomposites are presented in Figure 5b. It is clear that all the samples present a single decomposition step, with approximately 45% weight loss at 650 °C. The degradation of neat PEEK started at about 525 °C and shows the maximum decomposition temperature at 584 °C. The addition of 30 wt % CATAS reduced the thermal stability of the matrix to some extent: *T*_i_ and *T*_max_ decreased to 502 and 558 °C, respectively. Nevertheless, all the formulations presented a degradation curve similar to the matrix, with a superposition, in the thermal degradation pattern, because of the concomitant decomposition of the salts. The values of residual mass at the end of the tests, measured as 51% and 60% for neat PEEK and PEEK_30CATAS, respectively, are in line with the expected values, according to the registered weight loss for CATAS (43% at the end of the test) already measured in Figure 2d.

Dynamic mechanical thermal analysis was also used in order to evaluate the reinforcing effect of the CATAS on neat PEEK matrix. The storage modulus (G′) results, seen in Figure 5c clearly reveal that the presence of the fillers enhanced the modulus values with increasing content for all the studied samples. The adsorption of the CATAS onto the macromolecular chains of PEEK leads to a constraint in the chains movement and therefore, an improved storage modulus [23]. Results of the loss module (G”) from the DMTA tests can be seen in Figure 5d, where a moderate but progressive increase in the values as the filler content increases was observed, confirming the blocking effect of the CATAS also in the plastic behavior. The peaks in G” curves represent the middle temperature at which the materials are undergoing the maximum change in the mobility of the polymer chains, determining the glass transition temperature (*T*_g_). Regarding the *T*_g_ determined by DMTA, the matrix displayed a *T*_g_ of around 150 °C, while there was a slight variation in the glass transition temperatures for the other samples. This increase designates a restriction of the movement of the macromolecular chains of PEEK that contributes significantly to the stiffness of the materials as a result of enhanced interfacial interactions and conformational changes of the matrix in the vicinity of the nanofillers [24].

The role of CATAS on the viscoelastic behavior of PEEK was studied from the dynamic frequency sweep measurements at 400 °C. Profiles for complex viscosity, storage modulus, and loss modulus for neat PEEK and the nanocomposites are shown in Figure 5e,f. Pure PEEK showed the characteristic behavior of thermoplastic, Newtonian response in the complex viscosity at low frequency [25]. At low frequency values, the elastic modulus, G′, followed the expected behavior when polymer chains are fully relaxed, and then they presented initial stages of polymer-like terminal flow behavior. The addition of 10 wt % of CATAS had a limited effect. This nanocomposite kept the Newtonian behavior, but at low frequencies the complex viscosity began to increase slightly.

G′ at high frequency was practically unaffected due to a strong shear thinning behavior [26], and G” was similar in the whole frequency range, and consequently not important restrictions were expected in the polymer chain mobility. However, the increase of CATAS content to 20 and 30 wt % triggered changes in the rheological behavior of composites.

First, complex viscosity, G′, and G” rose in the whole frequency range analyzed. In the case of 30 wt %, at low frequency, the complex behavior began to leave the Newtonian behavior, because of the increase of the polymer chain mobility restrictions. This fact is due to the transition from liquid-like to solid-like viscoelastic behavior, caused by the particle–particle interactions that dominate over the polymer–filler interactions, forming an incipient interconnected network of CATAS [27,28]. In general, even large amounts of these nanofillers do not drastically change the rheological characteristics of the melt. This behavior suggests that use in massive quantities, or eventually together with other charges, is possible to reach significant amounts of charge to make heavily loaded composites.

The DMTA calculated G’ values have been reported in Table 2 and the percentage variation of the storage moduli has been calculated. From the table, it can be seen that at 100 °C the presence of nanoreinforcements produces an increase in the value of G’, which varies from 25% with 10% by weight of CATAS, an increase of about 48% with the intermediate amount of filler, up to an improvement of almost 60% for the PEEK-30CATAS formulation. The effect of nanofillers on G’ values measured at 200 °C is even more relevant. Above the PEEK *T*_g_, where the amorphous phase contributes in a reduced way to the mechanical properties, the G’ increments of the composites with the three percentages of CATAS are respectively 38%, 99%, and 115% with respect to the matrix. The noticeable improvement of G’ over the PEEK *T*_g_ suggests that there has been a variation involving the amorphous phase. Other possible causes of thermo-mechanical variations could be sought in the possible nucleating effect of the nanofillers that cause a variation of crystallinity degree. This network limits the large-scale motions of PEEK chains, affecting the global crystallization process, as is discussed below.

In order to better investigate this phenomenon, a thermal characterization with DSC instrument (first heating, cooling, and second heating scan cycle from 25 to 400 °C at 10 °C/min) was performed.

Cheng et al. studied the thermal properties of PEEK by DSC and demonstrated that a part of the amorphous phase of PEEK remains rigid over the glass transition temperature (*T*_g_) providing PEEK with a less flexible structure [29]. Similar results were observed from De Candia and Vittoria by analyzing PEEK membranes, likewise Huo and Cebe, and Kalika and Krishnaswamy reached similar conclusions studying the dielectric relaxation of PEEK [30,31,32]. They demonstrated that sometimes semicrystalline polymers show ∆*C*_p_ at *T*_g_ inconsistent with the amorphous fraction considered as a complement to 1 of the crystalline fraction (1 − *X*_c_). In fact, it is possible to calculate, with the variation of thermal capacity at *T*_g_, only a total rigid fraction (*X*_f_) which remains solid beyond the glass transition region. The overall rigid fraction (*X*_f_) consists of the crystalline fraction (*X*_c_) and another fraction, defined as a rigid amorphous fraction (*X*_raf_). Therefore, the rigid amorphous fraction (*X*_raf_) was included in the global rigid fraction (*X*_f_), since the rigid amorphous fraction cannot be detected by ∆*C*_p_ at *T*_g_.

In our specific case, the effect of CATAS presence on the three phases *X*_c_, *X*_a_, and *X*_raf_ in PEEK matrix was evaluated. The degree of crystallinity *X*_c_ was calculated using as a melting heat reference value for a 100% crystalline PEEK of Δ*H*^0^_m_ = 130 J/g [33,34]. The evaluation of the amorphous phase was made considering a value of the thermal capacity variation for a 100% amorphous PEEK ($∆C_{p}^{a}{}_{PEEK}$) of 0.350 J g^−1^ K^−1^. This value was obtained considering a not-amorphous phase *X*_fmin_ ≈ 0.12 at the maximum degree of quenching equal to Δ*C*_p max_ = 0.308 J g^−1^ K^−1^ for a PEEK quenched in liquid nitrogen [35]. We considered:

(2) $$X_{c}=\frac{∆H_{m}}{W_{PEEK}\times ∆H_{m}^{0}}$$

(3) $$X_{f}=1-\frac{W_{PEEK}\times ∆C_{p}{}_{PEEK}^{sc}}{∆C_{p}^{a}{}_{PEEK}}=1-\frac{∆C_{p}{}_{COMP}^{meas}-W_{CATAS}∆C_{p}{}_{CATAS}^{Tg}}{∆C_{p}^{a}{}_{PEEK}}\;\;=1-X_{a}$$

(4) $$X_{raf}=X_{f}-X_{c}$$

where *X*_c_ is the crystalline fraction, *W*_PEEK_ is the polymeric fraction in the composite, *X*_f_ is the blocked fraction (*X*_c_ + *X*_raf_) complement to 1 of the mobile amorphous fraction (maf) *X*_a_. *X*_raf_ is the rigid amorphous fraction. Thermal capacity of the semi-crystalline polymeric fraction of the composite $∆C_{p}{}_{PEEK}^{sc}$ is obtained from the difference between the measured heat capacity of the composite $∆C_{p}{}_{COMP}^{meas}$ and the thermal capacity of the filler in the same temperature range multiplied by its fraction, that is $W_{CATAS}∆C_{p}{}_{CATAS}^{Tg}$.

**Figure 6:**
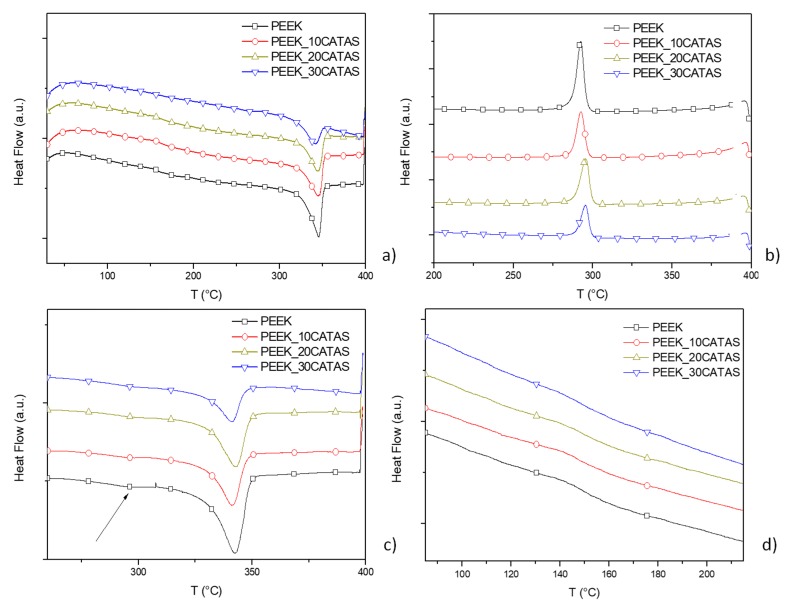
Differential scanning calorimetry (DSC) scans: (**a**) first heating; (**b**) cooling; (**c**) and (**d**) second heating scan (arrow for double melting peaks in (**c**) and zoom for *T*_g_ in (**d**)) of PEEK and PEEK composites at different CATAS content (symbols every 1000th points).

Figure 6 shows the results of first, cooling and second heating scans for neat PEEK matrix and for composites at different CATAS contents, while the results of the calculations for amorphous, crystalline and amorphous-rigid phases fractions are reported in Table 3.

Looking at the melting-crystallization overall behavior, a predictable variation for *T*_c_ and *T*_m_ was found in first heating and cooling scans (no substantial variation for *T*_m_ and slight reduction of *T*_c_ with increased CATAS content), while a double melting behavior was noted in the second heating (see arrow in the Figure 6c). The glass transition temperatures at the second heating scan are in the range 145–155 °C and increase slightly with the amount of salts. A negligible reduction of the *T*_g_ of the matrix is noted with respect to the first heating, which indicates a poor thermal degradation due to the test cycle. The composites do not show a detectable increase in *T*_g_ at the second heating, when compared to the first scan, probably because of the lower amount of amorphous phase, blocked in the RAF, and masked by the slight thermal degradation due to the test cycle. On the other hand, a change in the shape of the composites curves in the glass transition zone is detected at the second heating scan (reduced Δ*C*_p_ height and broadening of Δ*T* range), which is due to the reduced amount of matrix and decrease of the amorphous fraction in favor of RAF formation.

The melting behavior of PEEK has been already extensively described in the literature, attributing the double-melting behavior to partial melting and recrystallization of the lower melting lamellae during the DSC scan, formed additionally to higher melting ones. Lower melting crystallites can reorganize themselves to some extent during the DSC scan and eventually become part of the higher melting crystals at high temperature [36].

Regarding the different measured fractions, values in Table 3 show that, even at the first heating, where the thermal history related to the production of the material influences the crystallization kinetics, a more elevated fraction of the rigid amorphous phase with respect to the amorphous phase can be measured [37,38]. This trend is also confirmed for the crystallization during the cooling cycle, where it becomes more marked. The most consistent information is obtained from the fractions calculated for the second heating, where the trend is confirmed. A reduction in the amorphous phase, which becomes blocked, giving rise to a consistent fraction of a rigid amorphous phase, is observed (Figure 6d) [39]. It can be noted that, on the second heating scan, a substantial inversion of the relationship between *X*_a_ and *X*_raf_ is achieved, passing from PEEK to PEEK_30CATAS. The crystalline fraction undergoes a very slight increase with the addition of terephthalate salts, confirming a negligible nucleating effect of these fillers. The evaluation of phase fractions on composites with 10 and 20% of nanometric salts gave similar results, with lower *X*_raf_ fractions compared to PEEK_30CATAS [35].

## 4. Conclusions

Synthesized calcium terephthalate anhydrous nanosized salts were considered for the realization of nanocomposites at 10, 20, and 30 wt % in a PEEK matrix. Processability by melt compounding was empirically tested and confirmed with rheological tests. The DMTA characterization performed on the samples showed significant improvements up to 59% and to 116% of the storage module at 100 °C and 200 °C, respectively, for PEEK_30CATAS composite. The thermal study suggests that this type of nanofillers affects the PEEK phases with a negligible nucleating effect, slightly increasing the crystalline fraction. But above all, the CATAS have the effect of blocking a significant fraction of amorphous phase converting it into a more stable and performing rigid amorphous phase. The cost reduction is evident by estimating the filler price a few hundredths of the matrix price, thus obtaining a 20–25% decrease in the starting cost. Because of these positive results, it could be interesting to evaluate the effect of CATAS also together with the use of reinforcing fibers. We hypothesized that CATAS could have a synergistic effect if used together with other reinforcements, such as carbon or glass fibers. In fact, they could form a rigid amorphous phase around the reinforcing fibers as well as near the crystalline areas. This conformation would be able to distribute the tensions at the interface of the reinforced zone in a more progressive way up to the predominantly amorphous one, improving the thermomechanical performances of these hybrid composites. Upcoming activities are planned in this area of research.

## Figures and Tables

**Table 1:** Developed formulations based on poly (ether ether ketone) (PEEK) and calcium terephthalate anhydrous salts (CATAS).

Sample Name	PEEK wt %	CATAS wt %
PEEK	100	---
PEEK_10CATAS	90	10
PEEK_20CATAS	80	20
PEEK_30CATAS	70	30

**Table 2:** Calculated values for G’ at 100 and 200 °C for the PEEK/CATAS composites.

Sample Name	G’ [MPa] @100 °C	G’ [MPa] @200 °C	% ΔG’ 100 vs PEEK	% ΔG’ 200 vs PEEK
PEEK	1.03 × 10^9^	1.15 × 10^8^		
PEEK_10CATAS	1.29 × 10^9^	1.59 × 10^8^	+25.24	+38.26
PEEK_20CATAS	1.53 × 10^9^	2.29 × 10^9^	+48.54	+99.13
PEEK_30CATAS	1.64 × 10^9^	2.48 × 10^9^	+59.22	+115.65

**Table 3:** Evaluation of the phase fractions obtained from the DSC analysis of the PEEK matrix and composite with 30% of CATAS.

	Phase Fractions	PEEK	PEEK_30CATAS
First heating	Δ*H*_m_ (J/g)	47.5 ± 1.6	30.5 ± 2.3
	*X*_c_	36.6 ± 1.2	33.5 ± 2.6
	*X*_a_	47.9 ± 0.9	40.2 ± 0.8
	*X*_raf_	15.6 ± 0.3	26.4 ± 3.4
cooling	Δ*H*_c_ (J/g)	43.5 ± 2.1	32.2 ± 1.6
	*X*_c_	33.5 ± 1.6	35.4 ± 1.7
	*X*_a_	45.9 ± 0.6	12.9 ± 0.9
	*X*_raf_	20.6 ± 1.0	51.8 ± 2.6
Second heating	Δ*H*_m_ (J/g)	45.4 ± 1.3	32.4 ± 1.7
	*X*_c_	34.9 ± 1.0	35.6 ± 1.9
	*X*_a_	52.8 ± 1.1	16.9 ± 0.4
	*X*_raf_	12.3 ± 2.1	47.6 ± 1.5
